# Genome-Wide Association Study for Carcass Traits in an Experimental Nelore Cattle Population

**DOI:** 10.1371/journal.pone.0169860

**Published:** 2017-01-24

**Authors:** Rafael Medeiros de Oliveira Silva, Nedenia Bonvino Stafuzza, Breno de Oliveira Fragomeni, Gregório Miguel Ferreira de Camargo, Thaís Matos Ceacero, Joslaine Noely dos Santos Gonçalves Cyrillo, Fernando Baldi, Arione Augusti Boligon, Maria Eugênia Zerlotti Mercadante, Daniela Lino Lourenco, Ignacy Misztal, Lucia Galvão de Albuquerque

**Affiliations:** 1 School of Agricultural and Veterinarian Sciences, FCAV/ UNESP–Sao Paulo State University, Department of Animal Science, Jaboticabal-SP, Brazil; 2 University of Georgia, Department of Animal and Dairy Science, Athens, GA, United States of America; 3 APTA Center of Beef Cattle, Animal Science Institute, Sertaozinho, SP, Brazil; 4 Department of Animal Science, Federal University of Pelotas, Pelotas, RS, Brazil; Universita degli Studi di Bologna, ITALY

## Abstract

The purpose of this study was to identify genomic regions associated with carcass traits in an experimental Nelore cattle population. The studied data set contained 2,306 ultrasound records for *longissimus* muscle area (LMA), 1,832 for backfat thickness (BF), and 1,830 for rump fat thickness (RF). A high-density SNP panel (BovineHD BeadChip assay 700k, Illumina Inc., San Diego, CA) was used for genotyping. After genomic data quality control, 437,197 SNPs from 761 animals were available, of which 721 had phenotypes for LMA, 669 for BF, and 718 for RF. The SNP solutions were estimated using a single-step genomic BLUP approach (ssGWAS), which calculated the variance for windows of 50 consecutive SNPs and the regions that accounted for more than 0.5% of the additive genetic variance were used to search for candidate genes. The results indicated that 12, 18, and 15 different windows were associated to LMA, BF, and RF, respectively. Confirming the polygenic nature of the studied traits, 43, 65, and 53 genes were found in those associated windows, respectively for LMA, BF, and RF. Among the candidate genes, some of them, which already had their functions associated with the expression of energy metabolism, were found associated with fat deposition in this study. In addition, *ALKBH3* and *HSD17B12* genes, which are related in fibroblast death and metabolism of steroids, were found associated with LMA. The results presented here should help to better understand the genetic and physiologic mechanism regulating the muscle tissue deposition and subcutaneous fat cover expression of Zebu animals. The identification of candidate genes should contribute for Zebu breeding programs in order to consider carcass traits as selection criteria in their genetic evaluation.

## Introduction

Beef cattle production in tropical and subtropical regions is predominantly based on *Bos indicus* (Zebu) breeds and their crosses with *Bos taurus*. Even though Zebu animals have adaptive advantages to tropical conditions over Taurine breeds, these animals still have lower reproductive efficiency, lower beef tenderness, higher age at slaughtering, lower proportion of fat in the carcass [1,2]. The challenge for livestock production system in (sub) tropical countries is producing beef to satisfy consumer demands for quantity and quality and at the same time.

The *longissimus* muscle area (LMA) is an efficient indicator of the carcass yield, carcass muscularity [3], carcass weight, fat and muscle traits in steers [4]. The subcutaneous fat covering over the *longissimus* muscle acts minimizing evaporative weight loss of the carcass in the cooler besides been an efficient carcass-finishing indicative [5]. Furthermore, subcutaneous fat thickness is related to beef quality through protecting the muscle from cold-shortening which occur after the slaughter in the carcass cooling process. The conventional refrigeration of carcasses after slaughter may result in tougher meat, thus, an adequate quality carcass must have enough fat thickness to guarantee its preservation and desirable quality for consumption [6]. In addition, the beef cattle market specifications require a fat thickness higher than the usually produced by Zebu breeds. However, the low propensity to subcutaneous fat deposition is a problem in Zebu breeds, which have lower proportion of subcutaneous and intramuscular fat percentage than *Bos taurus* breeds [7, 8].

Favourable genetic correlations between subcutaneous fat and reproductive traits reported by Caetano et al. [9] indicated that high subcutaneous fat deposition could denote early finishing and result in sexually more precocious animals. On the other hand, undesirable genetic correlation between subcutaneous fat with weight gain and residual feed intake have been reported [9, 10] and caused some concerns in the authors.

The use of genomic information has been a standard procedure for genetic evaluation in animal and plant breeding programs in order to improve quantitative traits. This procedure has been especially helpful for traits that are hard or expensive to measure and consequently not routinely recorded. Genome-wide association studies (GWAS) aim to identify regions in the genome that are associated with phenotypes and have been applied in many traits including carcass traits [11]. A common method used for GWAS analyses is based on testing one marker at time as fixed effect. However, this approach assumes absence of population structure, which is very unlikely to happen when it is working on real data. An alternative to traditional GWAS consists to integrate all genotypic and phenotypic information available (from genotyped and ungenotyped animals) in one-step procedure (single-step GWAS) that allows the use of any model, and all relationships simultaneously [12].

Most of genomic studies for carcass traits have been applied on Taurine breeds in temperate regions [13]. Moreover, there is a need to study those traits in Zebu breeds under tropical conditions to elucidate the genetic architecture of carcass traits in these breeds. The purpose of this study was to identify associations between chromosomal regions with carcass traits such as *longissimus* muscle area (LMA), subcutaneous rump fat thickness (RF), and subcutaneous backfat thickness (BF) in an experimental Nelore cattle population.

## Material and Methods

### Data

The analysed Nelore cattle data set was provided by the APTA Beef Cattle Center—Institute of Animal Science (IZ), Sertãozinho, SP, Brazil. This herd has three experimental lines: a selection line (NeS) which has been selected for yearling weight since 1978; the traditional line (NeT) which has been submitted to the same selection criterion as NeS but, eventually, receives animals from other herds; and a control line (NeC) selected for average yearling weight (stabilizing selection). Ethics committee of the School of Agricultural and Veterinarian Sciences (FCAV), Sao Paulo State University (UNESP) and APTA Beef Cattle Center—Institute of Animal Science approved this study.

The data set contained pedigree information on 9,529 animals, of which 2,306 had ultrasound records for *longissimus* muscle area (LMA), 1,832 for backfat thickness (BF), and 1,830 rump fat thickness (RF). The ultrasound animals were born from 1996 to 2013. Table 1 shows the descriptive statistics for all the studied traits.

**Table 1:** Descriptive statistics for longissimus muscle area (LMA), backfat thickness (BF) and rump fat thicknes (RF) traits.

Traits	Mean (SD)		Np		Ngp	
	Male	Female	Male	Female	Male	Female
LMA(cm^2^)	53.59(8.86)	47.90(7.98)	1,384	922	471	250
BF(mm)	1.93(0.92)	2.54(1.49)	1,002	830	437	232
RF(mm)	4.40(1.84)	5.76(2.92)	916	914	469	249

SD = Standard deviation; Np = number of animals with phenotypic records; Ngp = number of genotyped animals with phenotypic records.

The herd management consisted on keeping the animals on pasture until seven months of age, when they were weaned. After this period, males and females (females born in 2004, 2005 and from 2008 to 2011) were submitted to a performance test on feedlot.

Serial ultrasound data were collected in vivo at 12 and 18 months of age for males and females, respectively. The difference among the age of phenotypic ultrasound measurement was mainly due the differed after weaning management applied for each sex. The animals were scanned by the PIE MEDICAL—Aquila equipment with probe of 7 inches and 3,5MHz, and then, the images were analysed using the Echo Image Viewer 1.0 (Pie Medical Equipament B.V., 1996) software. The probe was perpendicularly positioned between the 12th and 13th ribs on the left side to obtain the LMA and BF phenotypes. To obtain the RF phenotype, the probe was positioned at intersection of *Gluteus Medius* and *Biceps Femoris* muscles, which is located between ilium and ischium bones.

### Genotyping Procedure

DNA was extracted from blood samples which were collected in 9 ml K_2_EDTA Vacutainer blood collection tubes (BD Diagnostics) by jugular venepuncture. These samples were inverted to mix and prevent clotting, immediately placed in isothermic boxes, and transferred to the laboratory. During sampling animals were handled by qualified professionals who observed the procedure of well-being and safety of all animals.

A high-density SNP panel (BovineHD BeadChip assay 700k, Illumina Inc., San Diego, CA) was used for genotyping. SNP markers with minor allele frequency (MAF) and call rate less than 2% and 98%, respectively, were excluded. Also, samples with a call rate less than 90% were not considered in analyses. After genomic data quality control, 437,197 SNPs from 761 animals were available, of which 721 had phenotypes for LMA, 669 for BF, and 718 for RF trait (Table 1).

### (Co) Variance Component Estimation

The (co)variance components and genetic parameters were estimated by Bayesian inference, considering a linear single-trait animal model. Direct additive genetic and residual effects were included as random effects. Analyses were performed using GIBBS2f90 [14, 15]. The genetic and residual variance were obtained as follows:

$$var\left[\begin{array}{c} a\\ e \end{array}\right]=\left[\begin{array}{cc} H\sigma_{a}^{2} & 0\\ 0 & I\sigma_{a}^{2} \end{array}\right]$$ (1)

where σ^2^_a_ and σ^2^_e_ are total additive genetic and residual variances, respectively, **a** is the vector of direct additive genetic effects, **e** is a vector of residual effects, and **H** is a matrix that combines pedigree and genomic relationships, and its inverse consists on the integration of additive and genomic relationship matrices, **A** and **G**, respectively [15]:

$$H^{-1}=A^{-1}+\left[\begin{array}{cc} 0 & 0\\ 0 & G^{-1}-A_{22}^{-1} \end{array}\right],$$ (2)

Where **A**^**-1**^ is the inverse of relationship matrix based on pedigree information, **G**^**-1**^ is the inverse of genomic relationship matrix, which was constructed and scaled as described by VanRaden [16], and **A**^**-1**^_**22**_ is the inverse of pedigree-based relationship matrix for genotyped animals. The **H** matrix was built scaling **G** based on **A**_**22**_ considering that the average of diagonal of **G** is equal to average of the diagonal of **A**_**22**_ and average of off diagonal **G** is equal to average off diagonal **A**_**22.**_

**Fig 1:**
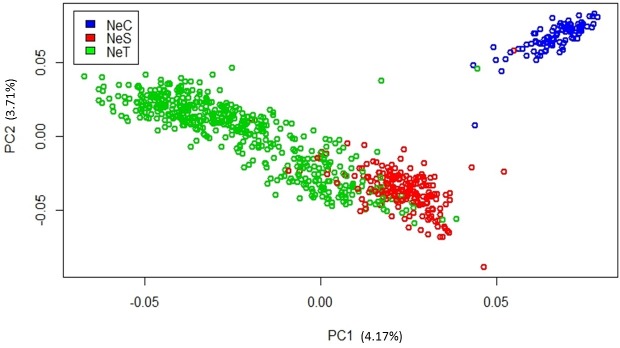
Distribution of animals provided by principal component.

The general model can be represented as follows:

Y=Xb+Za+e, (3)

where **Y** is the vector of phenotypic observations, **X** is an incidence matrix of phenotypes and fixed effects, **b** is the vector of fixed effects, that included contemporary groups (sex and year of birth), and age of ultrasound measurement as a covariable (linear and quadratic effects), **Z** is an incidence matrix that relates animals to phenotypes. The principal components analyses made based on genomic relationship matrix (**G)** revealed population sub-structure (Fig 1). Thus, the linear effect of the two principle components was considered in the model as covariable to correct for sub-structure of population as suggested by Price et al. [17].

Assumptions were:

E[y]=Xb,var[y]=ZƩZ′+R (4)

with Ʃ = var(a) = **H**σ^2^_a_ and **R** = **I**σ^2^_a_ in the single-trait model, where **H**σ^2^_a_ is the additive genetic variance and **I**σ^2^_e_ the residual variance, **H** is described above and **I** is the appropriate identity matrix. An inverted chi-square distribution was used for the prior values of the direct and residual genetic variances.

The posterior conditional distributions of **β**, **a**, and **e** effects were sampled from a multivariate normal distribution. The analysis consisted of a single chain of 300,000 cycles with a "burn-in" of 100,000 cycles, taking a sample every 10 iterations. Thus, 20,000 samples were used to obtain the parameters. Chain convergence was assessed by visual inspection. The posterior estimates were obtained retrospectively using the POSTGIBBSF90 program [14].

### Single Step Genome Wide Association Study (ssGWAS)

The analyses were performed using the single-step GWAS methodology [12] considering the same linear animal model used to estimate the (co) variance components before mentioned. The animal effects were decomposed in genotyped (**a**_**g**_) and ungenotyped (**a**_**n**_) animals as describe by Wang et al. [12], with the animal effect of genotyped animal:

ag=Zu, (5)

where **Z** was a matrix that related genotypes of each locus and **u** was a vector of marker effects. The variance of animal effects was assumed as:

$$var(a_{g})=var(Zu)=ZDZ’\sigma_{u}^{2}=G^{*}\sigma_{a}^{2},$$ (6)

where **D** was a diagonal matrix of weights for variances of markers (**D = I** for GBLUP), $\sigma_{u}^{2}$ was the genetic additive variance captured by each SNP marker when the weighted relationship matrix (G*) was built with no weight.

Thus, the SNP effects were obtained following equation, as described by Wang et al. [12]:

$$ȗ=\lambda DZ’\;G^{*-1}\;{â}_{g}={DZ’[ZDZ’]}^{-1}{â}_{g}$$ (7)

where *λ* was defined by VanRaden et al. [18] as a normalizing constant, as described below

$$\lambda =\frac{\sigma_{u}^{2}}{\sigma_{a}^{2}}=\frac{1}{\sum_{i=1}^{M}2p_{i}(1-p_{i})}$$ (8)

*M* was the number of SNPs and *p*_*i*_ was the frequency of the second allele in the *i*-th SNP.

The following iterative process described by Wang et al. [12] was used considering **D** to estimate the SNP effects: 1. **D** = **I**; 2. To calculate GEBVs for all animals in data set using ssGBLUP; 3. To calculate the SNP effect: **ȗ =** λDZ’G*^-1^**ȃ**_**g**_; 4. To calculate the variance of each SNP: d_*i*_ = û_*i*_^2^2*p*_*i*_ (1—*p*_*i*_), where *i* is the *i*-th marker; 5. To normalize the values of SNPs to keep constant the additive genetic variance; 6. To calculate the **G** matrix; 7. Exit, or loop to step 2.

The effects of markers were obtained by 2 iterations from step 2 to 6 as showed by Wang et al. [12]. The percentage of genetic variance explained by i-th region was calculated as described by Wang et al. [19]:

$$\frac{var(a_{i})}{\sigma_{a}^{2}}=x100\%=\frac{var(\sum_{j=i}^{50}z_{j}{û}_{j})}{\sigma_{a}^{2}}x100\%$$ (9)

where *a*_*i*_ is genetic value of the i-th region that consists of 50 consecutive SNPs, σ^2^_a_ is the total genetic variance, Z_j_ is vector of SNP content of the j-th SNP for all individuals, and û_j_ is marker effect of the *j*-th within the *i*-th region.

Analyses were performed using BLUPf90 family software [14] modified to include genomic information [15]. The results were presented by the proportion of variance explained by each window of 50 SNPs with average of 280 Kb.

### Search for Associated Genes

The chromosome segments that explained more than 0.5% of additive genetic variance were selected to explore and determine possible quantitative trait loci (QTL). The Map Viewer tool of bovine genome was used for identification of genes, available at "National Center for Biotechnology Information" (NCBI - http://www.ncbi.nlm.nih.gov) in UMD3.1 bovine genome assembly and Ensembl Genome Browser (http://www.ensembl.org/index.html). The classification of genes according to its biological function, identification of metabolic pathways and enrichment of genes was performed using the "Database for Annotation, Visualization and Integrated Discovery (DAVID) v. 6.7” (http://david.abcc.ncifcrf.gov/), GeneCards (http://www.genecards.org/), and UniProt (http://uniprot.org).

## Results

The posterior distribution for heritabilities estimated in this study are shown in Table 2. The known genes located in the associated windows are presented per each studied trait in Tables 3–5. A total of 12, 18 and 15 different windows were found associated to LMA, RF, and BF traits, respectively. In addition, many uncharacterized genes (LOC) were found in those regions associated to all studied traits. However, as mentioned by Fragomeni et al. [20], GWAS results should be carefully interpreted avoiding to determine an association as a causative effect since many QTL have been described for different traits but just a few of them have been validated by others studies. General information about all results of ssGWAS for all the studied traits are described in **S1–S3 Files**.

**Table 2:** Variance components, heritability estimates and their confidence interval for *longissimus* muscle area (LMA), backfat thickness (BF), and rump fat thickness (RF) carcass traits.

Traits	$\sigma_{a}^{2}$	$\sigma_{e}^{2}$	h^2^	95% Confidence Interval for h^2^	
	mean ± SD	mean ± SD	mean ±SD	CL	CU
LMA	17.94±2.29	19.80±1.56	0.47±0.05	0.44	0.51
BF	0.21±0.04	0.56±0.03	0.28±0.05	0.24	0.31
RF	0.80±0.14	1.75±0.11	0.31±0.05	0.28	0.35

$\sigma_{a}^{2}$ = additive genetic variance; $\sigma_{e}^{2}$ = residual variance; SD = standard deviation; h^2^ = heritability; CL, 95% = lower limit for 95% confidence intervals; CU, 95% = upper limit for 95% confidence intervals.

**Table 3:** Identification of genes based on additive genetic variance explained by windows of 50 adjacent SNPs for *longissimus* muscle area.

Chromosome	Position (bp)	Genes*	Var (%)
BTA1	5646552–5785986	*GRIK1; LOC101907301*	1.06
BTA1	4012541–4421333	*KRTAP7-1; LOC101906373; LOC101907866; LOC101907950; LOC785105*	0.51
BTA6	69231499–69368947	*CWH43; DCUN1D4; LOC101905687*	0.75
BTA7	89201585–89448769	*LOC104968974; RASA1; CCNH*	0.74
BTA8	31762825–31969601	*LOC782470; LOC104969317*	0.68
BTA14	80670924–80817593	*RALYL*	0.55
BTA15	74119235–74308370	*LOC104974311; API5*	0.61
BTA15	74571599–74924022	*HSD17B12; ALKBH3; C15H11orf96*	4.93
BTA15	76538448–76894000	*CHST1; LOC104974325; LOC104974324; SLC35C1; CRY2; MAPK8IP1; C15H11orf94; PEX16; GYLTL1B; PHF21A*	0.98
BTA21	21279610–21595190	*RLBP1; LOC104975343; TICRR; KIF7; PLIN1; PEX11A; WDR93;LOC104975344*	0.82
BTA24	48873432–49085371	*CTIF*	0.50
BTA28	33644873–33873100	*LOC101907374; DLG5*	0.89

*Official gene symbol (assembly UMD_3.1, annotation release 103); Var: Additive genetic variance explained by the 50 adjacent SNPs windows.

**Table 4:** Identification of genes based on additive genetic variance explained by windows of 50 SNPs for backfat thickness.

Chromosome	Position (bp)	Genes*	Var (%)
BTA1	27032026–27189437	*ARHGAP31; TMEM39A; POGLUT1; TIMMDC1; LOC104970865; CD80*	0.64
BTA2	99181462–99535059	*LOC101906862*	0.51
BTA2	119720331–119790546	*HTR2B; PSMD1; ARMC9*	1.35
BTA7	104518902–104847810	*LOC786544; GIN1; LOC101905525; PPIP5K2; LOC101905593; C7H5orf30; LOC786544*	0.70
BTA9	40405496–40651230	*METTL24*; *CDC40*; *WASF1*; *LOC104969538*	0.51
BTA10	41293230–41672195	*LOC104973133*	0.54
BTA11	26550217–26891283	*SLC3A1*; *LOC101906743*; *PREPL*; *CAMKMT*	0.52
BTA11	70031657–70122506	*LOC101905929*; *LOC104968430*	0.66
BTA14	21074886–21224382	*PRKDC*; *MCM4**; LOC100139903*	1.62
BTA14	22714764–22909901	*PCMTD1*; *LOC104974020*; *ST18*	0.70
BTA14	24376195–24543370	*XKR4*	0.61
BTA14	24874608–25102663	*LYN*; *RPS20*; *MOS*; *PLAG1*; *CHCHD7*	1.89
BTA14	25203669–25492467	*PENK*; *LOC101907667*	0.62
BTA16	60839844–61165403	*LOC100299281*; *LOC101902436*; *LOC104972944*; *MIR2285X*; *RASAL2*; *LOC101902850*	0.74
BTA21	6054709–6291692	*LOC104975304*; *LOC100301305*; *LOC101907131*; *ASB7*; *LINS*; *CERS3*	0.66
BTA29	46115170–46387810	*DOC2G*; *NUDT8*; *TBX10*; *LOC508879*; *UNC93B1*; *ALDH3B1*; *NDUFS8*; *TCIRG1*; *CHKA*; *SUV420H1*; *LOC104976291*	2.59

*Official gene symbol (assembly UMD_3.1, annotation release 103); Var: Additive genetic variance explained by the 50 SNPs windows.

**Table 5:** Identification of genes based on additive genetic variance explained by windows of 50 SNPs for rump fat thickness.

Chromosome	Position (bp)	Genes*	Var (%)
BTA2	33484646–33713607	*KCNH7*	1.19
BTA2	34215251–34433259	*IFIH1*; *FAP*; *GCG*	0.65
BTA2	50272472–50597681	*LOC510454*; *LOC100295230*	0.96
BTA2	106280299–106622973	*TRNAC-ACA*	1.27
BTA5	20993029–21117110	*KERA*; *TRNAC-ACA*; *LUM*; *DCN*	1.70
BTA6	53086533–53397963	*LOC100139828*; *LOC788223*	0.79
BTA6	54794939–54953177	*LOC104968862*; *LOC104968863*	1.22
BTA6	55345470–55570440	*LOC100296974*; *LOC100296505*; *LOC104968886*	1.27
BTA9	8440022–8597025	*BAI3*	1.03
BTA13	69726309–69962829	*LOC104973877*	0.74
BTA14	24877166–25105265	*LYN*; *RPS20*; *MOS*; *PLAG1*; *CHCHD7*; *SDR16C5*	2.38
BTA14	25203669–25492467	*PENK*; *LOC101907667*	1.93
BTA15	57005358–57277927	*LOC104974276*; *ACER3*; *LOC786726*; *LOC104974277*; *B3GNT6*	1.00
BTA15	57296895–57435696	*CAPN5*; *MYO7A*; *LOC786996*	2.28
BTA19	10837090–11303460	*CLTC*; *PTRH2*; *VMP1*; *LOC104974991*; *MIR21*; *LOC104968960*; *TUBD1*; *RPS6KB1*;*RNFT1*; *LOC101907104*	2.41
BTA19	31689778–31937809	*MYOCD*; *LOC104975039*; *ARHGAP44*	0.58
BTA19	32305077–32647787	*HS3ST3A1*; *LOC104975040*	0.61
BTA20	16231525–16430411	*LOC104975227*	0.60

*Official gene symbol (assembly UMD_3.1, annotation release 103); Var: Additive genetic variance explained by the 50 SNPs windows.

**Fig 2:**
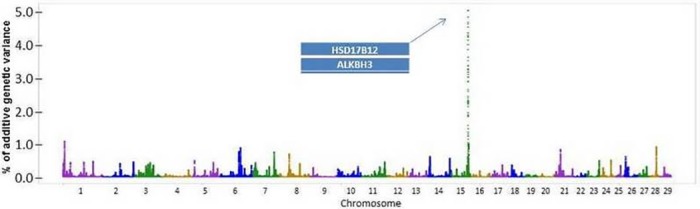
Additive genetic variance explained by windows of 50 adjacent SNPs distributed by chromosomes for *longissimus* muscle area.

A total of 28 known genes and 15 uncharacterized genes were found associated to LMA (**Fig 2** and **Table 3**). The first cluster from enrichment analysis for LMA was related to processes that stop, prevent or reduce the rate of cell death by apoptotic process (S1 Table). In addition, the second cluster was related to DNA repair, mechanisms that minimize acute damage to the cell's overall integrity and response to DNA damage stimulus. Both processes are dependent on each other, because failures in DNA repair will increase the rate of cell death.

**Fig 3:**
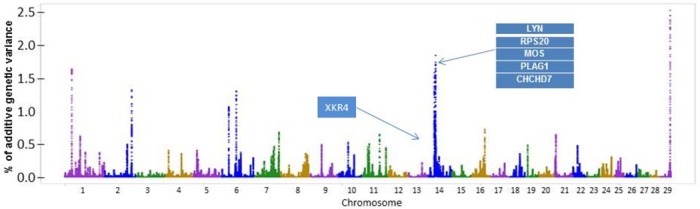
Additive genetic variance explained by windows of 50 adjacent SNPs distributed by chromosomes for backfat thickness in Nelore.

A total of 42 known genes and 23 uncharacterized genes (located on BTA1, BTA2, BTA7, BTA9, BTA10, BTA11, BTA14, BTA16, BTA21, and BTA29) were found in those windows associated to BF (**Fig 3 and Table 4**). The clusters obtained by the genes enrichment analysis for BF were related to ATP-binding, transcription regulation, and protein amino acid phosphorylation (**S2 Table**). Many genes that are related to lipids and fat expression were found in the windows associated with BF.

**Fig 4:**
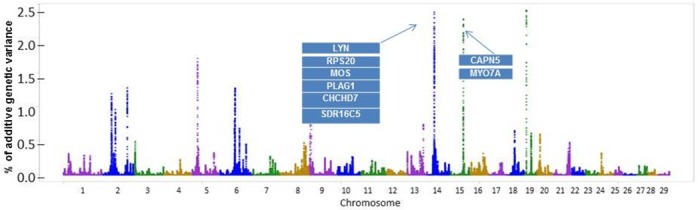
Additive genetic variance explained by windows of 50 adjacent SNPs distributed by chromosomes for rump fat thickness.

A total of 32 known genes and 19 uncharacterized genes (located in BTA2, BTA5, BTA6, BTA9, BTA13, BTA14, BTA15, BTA19, and BTA20) were found in the windows associated to RF (**Fig 4**). Some of those regions have been reported to be associated with lipid production (**Table 5**). These findings confirm the polygenic nature of fat deposition. The clusters obtained by the genes enrichment analysis of RF are related to ATP binding, response to hormone stimulus and intracellular signalling cascade (**S3 Table**).

A window located on BTA14 (at 24.87–25.11 Mb) was associated to both fat deposition traits, BF and RF, where the *LYN*, *RPS20*, *MOS*, *PLAG1*, and *CHCHD7* genes are located.

## Discussion

The confidence interval for posterior distribution of heritability was very accurate for all studied traits; the range was always within 4 points from the mean. This narrow posterior distribution indicates a good adjustment of the **H** matrix to the structure of analysed population. Heritability values showed that a great part of phenotypic variance was due to additive genetic effects. Thus, it is important to identify the genes involved in these traits expression, especially major effects genes.

Several known genes that have their functions previously reported associated to the expression of energy metabolism were confirmed associated with fat deposition in this study. In addition, genes such as the *ALKBH3* and *HSD17B12*, which are related in fibroblast death and metabolism of steroids, were found associated with *longissimus* muscle area.

The *ALKBH3* gene (found in a window that most explained additive genetic variance of LMA located in BTA15) codifies an intrinsic DNA repair protein that suppresses transcription associated DNA damage at highly expressed genes [21]. Even though this gene has not been associated for those traits in a cattle population, Nay et al. [22] reported that deleting it in mice made fibroblasts more susceptible to death. Still in this same associated window, the *HSD17B12* gene is located. This gene is a member of the hydroxysteroid dehydrogenase superfamily, involved in the metabolism of steroids, retinoids, bile and fatty acids that apparently can be related in metabolic pathways involved in tumor [23]. Considering that tumor results from uncontrolled cells multiplication, it could be related to DNA repair.

The *HTR2B* gene, located on BTA2 (**Table 4**), was found in a window associated to BF. This gene has been previously associated to fat deposition in humans [24] and it is known that it encodes one of the several different receptors for 5-hydroxytryptamine (serotonin). The serotonin hormone acts as a neurotransmitter, a hormone, and a mitogen. Two candidate genes were found in the window that explained the greatest part of genetic variance (BTA29): *ALDH3B1* and *CHKA* genes. The *ALDH3B1* gene had its function related to oxidation of lipid-derived aldehydes generated in the human plasma membrane [25] and is associated to diabetes in humans [26]. The biological process of the *CHKA* gene is related to lipid biosynthesis and it has been reported differentially regulated in mice by high fatty acids levels [27]. Even though some genes found in the associated windows have not been directly associated to the expression of these traits in previous researches, it is important to discuss a bit about their functions.

The *XKR4* gene (placed in a window associated with BF located on BTA14) was previously reported by Porto Neto et al. [27] to be associated with RF in a recent genome-wide association study in Belmont Red and Santa Gertrudis breeds. The authors found association between RF and three SNPs within *XKR4* gene (P < 0.001), one of the SNPs alone explained 5.9% of the additive genetic variance. This gene has also been reported as a candidate gene for residual feed intake, average daily feed intake and average daily gain [28]. In addition, Bastion et al. [29] suggested the *XKR4* gene participates in the regulation of prolactin secretion in cattle.

A window located on BTA14 (at 24.87–25.11 Mb) was associated to both fat deposition traits, BF and RF (**Table 4** and **Table 5**). In this associated window were found the *PLAG1*, *CHCHD7*, *MOS*, *RPS20*, *LYN*, and *RDHE2* (also known as *SDR16C5*) genes. There are evidences that these genes are directly associated with the expression of many traits in beef cattle. Independent studies have reported association between *PLAG1* gene with fat, residual feed intake, carcass, performance and reproductive traits in beef cattle breeds including Nelore, Japanese Black and Korean cattle [30–35]. The *PENK* gene, also located in a window on BTA14 (at 25.20–25.49 Mb), was previously reported associated with residual feed intake in a swine population [36]. The windows that these two genes, *PENK* and *PLAG1*, are located (at 24.87–25. 10 Mb and 25.20–25.49 Mb of BTA15) explained a great part of additive genetic variance of both fat deposition traits, BF and RF. Thus, once they are juxtaposed and with possible physical disequilibrium, it is hard to say whether these genes have independent function or the associated windows where they are located are in linkage disequilibrium. Karim et al. [37] reported that causative mutation in the *PLAG1***-***CHCHD7* intergenic region affected the bovine stature in a European breed. Nishimura et al. [30] re-exanimated this region in a Japanese Black cattle founding three QTL for carcass weight and strongly suggested that it was identical to the causative variation reported by Karim et al. [37]. There are evidences that these genes have influence on human height [38, 39].

Even for similar traits such as BF and RF, which are highly phenotypic (0.61) and genetic (0.79 ± 0.05) correlated [40] differing in the site of fat deposition, they cannot be considered the same trait. Thus, differences in the genomic regions that influence these traits were found here, which might be due epigenetic effect that may regulate them. Li et al. [41] concluded that methylation differences between adipose depots are dependent on their location. The genes that act depositing lipids in several regions are not always the same, the methylation depends on the region where these genes are located.

Genes related with fat deposition were found in windows that explained a great part additive genetic variance of RF trait. The *CAPN5* and *MYO7A* (located at 57.29–57.43 Mb of BTA15) genes are associated with blood cholesterol level in humans and with abdominal fat in chicken [42, 43]. In addition, the *LUM* gene (located at 20.99–21.12 Mb of BTA5) was related with omental fat deposition in humans [44]. The *BAI3* gene, placed in a region of BTA9 acts on the fusion of myoblast and it is expressed on extracellular matrix [45] that participates in the formation of adipose tissue.

Some others genes had their expression identified in skeletal muscle and performance capability on adipose tissue, since that tissue are juxtaposed and have many genes with common acting. According to Wang et al. [46], the *RPS6KB1* gene (located at 10.84–11.30 Mb of BTA19) is sub expressed in skeletal muscle of calves which were experimental fed with high levels of protein. In addition, the *DCN* gene (located at 20.99–21.12 Mb of BTA5) operates in intramyocellular space co-acting with myostatin gene [47] and it is in extracellular matrix with highly potential to contribute for fat deposition [48].

Considering that *longissimus* muscle area, backfat thickness and rump fat thickness traits are governed by many variants with small effects, the results in the present study confirm the polygenic effect of these traits. On the other hand, it is important to highlight that while several DNA regions with larger effects have been detected, some of these may not be due to variants but could be mark chromosome segments from important ancestors or just be sampling noise. In this study, 437,197 SNPs effects were predicted from about 700 data points (DGVs). With so few points, there is a statistical possibility that a limited number of SNP could provide good prediction as shown by cross-validation just by chance. Stam [49] pointed out that the number of independent chromosome segments due to small effective population size is small. Therefore, it is possible that GWAS as performed here may have a limited resolution and could be subjected to high sampling noise. However, our results are very consistent and many genomic regions with known genes that have their functions already described were confirmed associate to the studied traits. Besides the known genes, many uncharacterized genes were found in the associated windows. In this manner, information about the associations found in this study might be used in future studies with the intention of characterization of those genes. Such studies would be used to confirm the association between genes in those chromosomic regions and carcass traits.

The results presented here should help to better understand the genetic and physiologic mechanism regulating the muscle tissue deposition and subcutaneous fat cover expression of Zebu animals. The analysed data set contained information from proved animals of an experimental herd, which have progenies participating in many breeding programs in several regions of Brazil. Despite the polygenic nature of the studied traits, some genes found in the associated windows, such as *ALKBH3* and *HSD17B1*2 are more likely to be related to *longissimus* muscle area, as well as the *PLAG1*, *CAPN5*, *MYO7A* and *XKR4* genes, which are possibly associated with fat deposition in Nelore cattle. Thus, the identification of these genes should contribute for future studies to validate them to use as candidate genes for Zebu breeding programs.

## Supporting Information

S1 File Results of ssGWAS for *longissimus* muscle area. (XLSX)

S2 File Results of ssGWAS for back fat thickness. (XLSX)

S3 File Results of ssGWAS for rump fat thickness. (XLSX)

S1 Table Gene enrichment clustering for *longissimus* muscle area. (DOCX)

S2 Table Gene enrichment clustering for backfat thickness. (DOC)

S3 Table Gene enrichment clustering for rump fat thickness. (DOCX)
