# Supplementary material for: Genome-Wide Association Study for Carcass Traits in an Experimental Nelore Cattle Population
Source: PLoS One. 2017 Jan 24;12(1):e0169860. doi: 10.1371/journal.pone.0169860 (PMC5261778; doi:10.1371/journal.pone.0169860)
Supplement: S1 Table — (DOCX) [file pone.0169860.s004.docx]

S1 Table. Gene enrichment clustering for *longissimus* muscle area

| Annotation Cluster 1 | | | | Enrichment Score: 1.13 | | | |
| --- | --- | --- | --- | --- | --- | --- | --- |
| Category | | Term | | Count | % | PValue | FDR |
| SMART | | SM00326:SH3 | | 3 | 1.31 | 0.01 | 9.69 |
| UP_SEQ_FEATURE | | domain:SH3 | | 3 | 1.31 | 0.02 | 18.83 |
| SP_PIR_KEYWORDS | | sh3 domain | | 3 | 1.31 | 0.03 | 24.38 |
| INTERPRO | | IPR001452:Src homology-3 domain | | 3 | 1.31 | 0.03 | 23.58 |
| GOTERM_BP_FAT | | GO:0043066~negative regulation of apoptosis | | 3 | 1.31 | 0.09 | 70.10 |
| GOTERM_BP_FAT | | GO:0043069~negative regulation of programmed cell death | | 3 | 1.31 | 0.09 | 71.01 |
| GOTERM_BP_FAT | | GO:0060548~negative regulation of cell death | | 3 | 1.31 | 0.09 | 71.19 |
| GOTERM_BP_FAT | | GO:0042981~regulation of apoptosis | | 4 | 1.75 | 0.10 | 75.11 |
| GOTERM_BP_FAT | | GO:0043067~regulation of programmed cell death | | 4 | 1.75 | 0.10 | 75.94 |
| GOTERM_BP_FAT | | GO:0010941~regulation of cell death | | 4 | 1.75 | 0.10 | 76.24 |
| SP_PIR_KEYWORDS | | cytoplasm | | 6 | 2.62 | 0.37 | 99.25 |
| GOTERM_BP_FAT | | GO:0007242~intracellular signaling cascade | | 3 | 1.31 | 0.54 | 100.00 |
|  | |  | |  |  |  |  |
| Annotation Cluster 2 | | | | Enrichment Score: 0.93 | | | |
| Category | | Term | | Count | % | PValue | FDR |
| GOTERM_BP_FAT | | GO:0033554~cellular response to stress | | 4 | 1.75 | 0.04 | 44.01 |
| GOTERM_BP_FAT | | GO:0006281~DNA repair | | 3 | 1.31 | 0.06 | 55.54 |
| GOTERM_BP_FAT | | GO:0006974~response to DNA damage stimulus | | 3 | 1.31 | 0.10 | 73.44 |
| GOTERM_BP_FAT | | GO:0006259~DNA metabolic process | | 3 | 1.31 | 0.16 | 89.64 |
| SP_PIR_KEYWORDS | | nucleus | | 6 | 2.62 | 0.61 | 100.00 |
|  | |  | |  |  |  |  |
| Annotation Cluster 3 | | | | Enrichment Score: 0.44 | | | |
| Category | | | Term | Count | % | PValue | FDR |
| GOTERM_BP_FAT | | | GO:0019725~cellular homeostasis | 3 | 1.31 | 0.14 | 85.98 |
| UP_SEQ_FEATURE | | | transmembrane region | 9 | 3.93 | 0.22 | 93.21 |
| SP_PIR_KEYWORDS | | | transmembrane | 9 | 3.93 | 0.22 | 93.38 |
| GOTERM_BP_FAT | | | GO:0042592~homeostatic process | 3 | 1.31 | 0.28 | 98.82 |
| SP_PIR_KEYWORDS | | | membrane | 10 | 4.37 | 0.32 | 98.32 |
| UP_SEQ_FEATURE | | | topological domain:Cytoplasmic | 6 | 2.62 | 0.38 | 99.48 |
| GOTERM_CC_FAT | | | GO:0016021~integral to membrane | 9 | 3.93 | 0.49 | 99.91 |
| GOTERM_CC_FAT | | | GO:0031224~intrinsic to membrane | 9 | 3.93 | 0.54 | 99.97 |
| UP_SEQ_FEATURE | | | glycosylation site:N-linked (GlcNAc...) | 5 | 2.18 | 0.76 | 100.00 |
| SP_PIR_KEYWORDS | | | glycoprotein | 5 | 2.18 | 0.79 | 100.00 |
| Annotation Cluster 4 | | | | Enrichment Score: 0.21 | | | |
| Category | Term | | | Count | % | PValue | FDR |
| GOTERM_CC_FAT | GO:0005654~nucleoplasm | | | 3 | 1.31 | 0.36 | 99.04 |
| SP_PIR_KEYWORDS | nucleus | | | 6 | 2.62 | 0.61 | 100.00 |
| GOTERM_CC_FAT | GO:0031981~nuclear lumen | | | 3 | 1.31 | 0.62 | 100.00 |
| GOTERM_CC_FAT | GO:0070013~intracellular organelle lumen | | | 3 | 1.31 | 0.74 | 100.00 |
| GOTERM_CC_FAT | GO:0043233~organelle lumen | | | 3 | 1.31 | 0.75 | 100.00 |
| GOTERM_CC_FAT | GO:0031974~membrane-enclosed lumen | | | 3 | 1.31 | 0.76 | 100.00 |
|  |  | | |  |  |  |  |
| Annotation Cluster 5 | | | | Enrichment Score: 0.17 | | | |
| Category | Term | | | Count | % | PValue | FDR |
| GOTERM_BP_FAT | GO:0051252~regulation of RNA metabolic process | | | 4 | 1.75 | 0.48 | 99.98 |
| SP_PIR_KEYWORDS | nucleus | | | 6 | 2.62 | 0.61 | 100.00 |
| SP_PIR_KEYWORDS | transcription regulation | | | 3 | 1.31 | 0.71 | 100.00 |
| SP_PIR_KEYWORDS | Transcription | | | 3 | 1.31 | 0.73 | 100.00 |
| GOTERM_BP_FAT | GO:0006355~regulation of transcription, DNA-dependent | | | 3 | 1.31 | 0.73 | 100.00 |
| GOTERM_BP_FAT | GO:0045449~regulation of transcription | | | 4 | 1.75 | 0.74 | 100.00 |
| GOTERM_BP_FAT | GO:0006350~transcription | | | 3 | 1.31 | 0.82 | 100.00 |
|  |  | | |  |  |  |  |
| Annotation Cluster 6 | | | | Enrichment Score: 0.07 | | | |
| Category | Term | | | Count | % | PValue | FDR |
| SP_PIR_KEYWORDS | cell membrane | | | 3 | 1.31 | 0.76 | 100.00 |
| GOTERM_CC_FAT | GO:0044459~plasma membrane part | | | 3 | 1.31 | 0.84 | 100.00 |
| GOTERM_CC_FAT | GO:0005886~plasma membrane | | | 4 | 1.75 | 0.94 | 100.00 |

FDR = False Discovery Ratio
