# Supplementary material for: Genome-Wide Association Study for Carcass Traits in an Experimental Nelore Cattle Population
Source: PLoS One. 2017 Jan 24;12(1):e0169860. doi: 10.1371/journal.pone.0169860 (PMC5261778; doi:10.1371/journal.pone.0169860)
Supplement: S2 Table — (DOC) [file pone.0169860.s005.doc]

S2 Table. Gene enrichment clustering for backfat thickness.

| Annotation Cluster 1 | | Enrichment Score: 0.90 | | | |
| --- | --- | --- | --- | --- | --- |
| Category | Term | Count | % | PValue | FDR |
| SP_PIR_KEYWORDS | phosphotransferase | 3 | 0.83 | 0.02 | 18.47 |
| SP_PIR_KEYWORDS | transferase | 7 | 1.93 | 0.03 | 24.82 |
| SP_PIR_KEYWORDS | kinase | 5 | 1.38 | 0.03 | 28.14 |
| SP_PIR_KEYWORDS | atp-binding | 6 | 1.66 | 0.06 | 50.13 |
| GOTERM_MF_FAT | GO:0005524~ATP binding | 6 | 1.66 | 0.10 | 67.07 |
| GOTERM_MF_FAT | GO:0032559~adenyl ribonucleotide binding | 6 | 1.66 | 0.10 | 68.56 |
| GOTERM_MF_FAT | GO:0030554~adenyl nucleotide binding | 6 | 1.66 | 0.12 | 74.74 |
| GOTERM_MF_FAT | GO:0001883~purine nucleoside binding | 6 | 1.66 | 0.12 | 75.74 |
| GOTERM_MF_FAT | GO:0001882~nucleoside binding | 6 | 1.66 | 0.12 | 76.49 |
| GOTERM_BP_FAT | GO:0016310~phosphorylation | 4 | 1.10 | 0.13 | 84.82 |
| SP_PIR_KEYWORDS | nucleotide-binding | 6 | 1.66 | 0.13 | 79.45 |
| GOTERM_MF_FAT | GO:0032553~ribonucleotide binding | 6 | 1.66 | 0.19 | 90.20 |
| GOTERM_MF_FAT | GO:0032555~purine ribonucleotide binding | 6 | 1.66 | 0.19 | 90.20 |
| GOTERM_BP_FAT | GO:0006793~phosphorus metabolic process | 4 | 1.10 | 0.19 | 94.53 |
| GOTERM_BP_FAT | GO:0006796~phosphate metabolic process | 4 | 1.10 | 0.19 | 94.53 |
| GOTERM_MF_FAT | GO:0017076~purine nucleotide binding | 6 | 1.66 | 0.21 | 92.93 |
| UP_SEQ_FEATURE | nucleotide phosphate-binding region:ATP | 4 | 1.10 | 0.24 | 96.10 |
| GOTERM_MF_FAT | GO:0004672~protein kinase activity | 3 | 0.83 | 0.27 | 96.69 |
| UP_SEQ_FEATURE | binding site:ATP | 3 | 0.83 | 0.27 | 97.66 |
| GOTERM_BP_FAT | GO:0006468~protein amino acid phosphorylation | 3 | 0.83 | 0.31 | 99.36 |
| GOTERM_MF_FAT | GO:0000166~nucleotide binding | 6 | 1.66 | 0.32 | 98.61 |
| Annotation Cluster 2 | | Enrichment Score: 0.45 | | | |
| Category | Term | Count | % | PValue | FDR |
| GOTERM_MF_FAT | GO:0003677~DNA binding | 6 | 1.66 | 0.19 | 89.57 |
| SP_PIR_KEYWORDS | transcription regulation | 5 | 1.38 | 0.26 | 96.35 |
| SP_PIR_KEYWORDS | Transcription | 5 | 1.38 | 0.35 | 99.08 |
| GOTERM_BP_FAT | GO:0006350~transcription | 5 | 1.38 | 0.38 | 99.85 |
| SP_PIR_KEYWORDS | dna-binding | 4 | 1.10 | 0.42 | 99.77 |
| SP_PIR_KEYWORDS | nucleus | 8 | 2.21 | 0.46 | 99.90 |
| GOTERM_BP_FAT | GO:0045449~regulation of transcription | 5 | 1.38 | 0.57 | 100.00 |
| Annotation Cluster 3 | | Enrichment Score: 0.04 | | | |
| Category | Term | Count | % | PValue | FDR |
| SP_PIR_KEYWORDS | metal-binding | 4 | 1.10 | 0.85 | 100.00 |
| GOTERM_MF_FAT | GO:0046914~transition metal ion binding | 4 | 1.10 | 0.86 | 100.00 |
| GOTERM_MF_FAT | GO:0043169~cation binding | 5 | 1.38 | 0.94 | 100.00 |
| GOTERM_MF_FAT | GO:0043167~ion binding | 5 | 1.38 | 0.94 | 100.00 |
| GOTERM_MF_FAT | GO:0046872~metal ion binding | 4 | 1.10 | 0.98 | 100.00 |
| Annotation Cluster 4 | | Enrichment Score: 0.01 | | | |
| Category | Term | Count | % | PValue | FDR |
| SP_PIR_KEYWORDS | glycoprotein | 4 | 1.10 | 0.96 | 100.00 |
| UP_SEQ_FEATURE | transmembrane region | 5 | 1.38 | 0.97 | 100.00 |
| UP_SEQ_FEATURE | glycosylation site:N-linked (GlcNAc...) | 4 | 1.10 | 0.97 | 100.00 |
| SP_PIR_KEYWORDS | membrane | 6 | 1.66 | 0.98 | 100.00 |
| SP_PIR_KEYWORDS | transmembrane | 5 | 1.38 | 0.99 | 100.00 |
| GOTERM_CC_FAT | GO:0016021~integral to membrane | 5 | 1.38 | 1.00 | 100.00 |
| GOTERM_CC_FAT | GO:0031224~intrinsic to membrane | 5 | 1.38 | 1.00 | 100.00 |
