# Supplementary material for: Genome-Wide Association Study for Carcass Traits in an Experimental Nelore Cattle Population
Source: PLoS One. 2017 Jan 24;12(1):e0169860. doi: 10.1371/journal.pone.0169860 (PMC5261778; doi:10.1371/journal.pone.0169860)
Supplement: S3 Table — (DOCX) [file pone.0169860.s006.docx]

S3 Table. Gene enrichment clustering for rump fat thickness

| Annotation Cluster 1 | | Enrichment Score: 1.29 | | | |
| --- | --- | --- | --- | --- | --- |
| Category | Term | Count | % | PValue | FDR |
| SP_PIR_KEYWORDS | proteoglycan | 3 | 0.98 | 0.00 | 2.56 |
| UP_SEQ_FEATURE | repeat:LRR 12 | 3 | 0.98 | 0.01 | 7.20 |
| UP_SEQ_FEATURE | repeat:LRR 11 | 3 | 0.98 | 0.01 | 8.91 |
| INTERPRO | IPR000372:Leucine-rich repeat, cysteine-rich flanking region, N-terminal | 3 | 0.98 | 0.01 | 9.13 |
| SMART | SM00013:LRRNT | 3 | 0.98 | 0.01 | 8.07 |
| UP_SEQ_FEATURE | repeat:LRR 10 | 3 | 0.98 | 0.01 | 11.45 |
| UP_SEQ_FEATURE | repeat:LRR 9 | 3 | 0.98 | 0.01 | 14.79 |
| UP_SEQ_FEATURE | repeat:LRR 8 | 3 | 0.98 | 0.02 | 17.57 |
| UP_SEQ_FEATURE | compositionally biased region:Cys-rich | 3 | 0.98 | 0.02 | 19.42 |
| UP_SEQ_FEATURE | repeat:LRR 7 | 3 | 0.98 | 0.02 | 23.49 |
| UP_SEQ_FEATURE | repeat:LRR 6 | 3 | 0.98 | 0.03 | 30.86 |
| UP_SEQ_FEATURE | repeat:LRR 5 | 3 | 0.98 | 0.04 | 35.64 |
| INTERPRO | IPR001611:Leucine-rich repeat | 3 | 0.98 | 0.04 | 33.60 |
| SP_PIR_KEYWORDS | extracellular matrix | 3 | 0.98 | 0.04 | 36.90 |
| UP_SEQ_FEATURE | repeat:LRR 4 | 3 | 0.98 | 0.04 | 40.41 |
| UP_SEQ_FEATURE | repeat:LRR 3 | 3 | 0.98 | 0.06 | 50.75 |
| UP_SEQ_FEATURE | repeat:LRR 1 | 3 | 0.98 | 0.06 | 54.72 |
| UP_SEQ_FEATURE | repeat:LRR 2 | 3 | 0.98 | 0.06 | 54.92 |
| SP_PIR_KEYWORDS | leucine-rich repeat | 3 | 0.98 | 0.07 | 54.29 |
| GOTERM_CC_FAT | GO:0005578~proteinaceous extracellular matrix | 3 | 0.98 | 0.08 | 61.75 |
| GOTERM_CC_FAT | GO:0031012~extracellular matrix | 3 | 0.98 | 0.09 | 66.71 |
| UP_SEQ_FEATURE | disulfide bond | 7 | 2.30 | 0.15 | 86.00 |
| SP_PIR_KEYWORDS | disulfide bond | 7 | 2.30 | 0.17 | 88.37 |
| SP_PIR_KEYWORDS | Secreted | 5 | 1.64 | 0.17 | 88.86 |
| GOTERM_CC_FAT | GO:0005576~extracellular region | 5 | 1.64 | 0.35 | 99.26 |
| SP_PIR_KEYWORDS | signal | 6 | 1.97 | 0.42 | 99.82 |
| GOTERM_CC_FAT | GO:0044421~extracellular region part | 3 | 0.98 | 0.42 | 99.81 |
| UP_SEQ_FEATURE | signal peptide | 6 | 1.97 | 0.42 | 99.86 |
| GOTERM_BP_FAT | GO:0007186~G-protein coupled receptor protein signaling pathway | 3 | 0.98 | 0.56 | 100.00 |
| GOTERM_BP_FAT | GO:0007166~cell surface receptor linked signal transduction | 4 | 1.31 | 0.60 | 100.00 |
| Annotation Cluster 2 | | Enrichment Score: 0.98 | | | |
| Category | Term | Count | % | PValue | FDR |
| GOTERM_BP_FAT | GO:0050953~sensory perception of light stimulus | 3 | 0.98 | 0.05 | 49.31 |
| GOTERM_BP_FAT | GO:0007601~visual perception | 3 | 0.98 | 0.05 | 49.31 |
| GOTERM_BP_FAT | GO:0050890~cognition | 5 | 1.64 | 0.06 | 55.39 |
| GOTERM_BP_FAT | GO:0050877~neurological system process | 5 | 1.64 | 0.13 | 85.21 |
| GOTERM_BP_FAT | GO:0007600~sensory perception | 4 | 1.31 | 0.14 | 88.13 |
| SP_PIR_KEYWORDS | Secreted | 5 | 1.64 | 0.17 | 88.86 |
| GOTERM_CC_FAT | GO:0005576~extracellular region | 5 | 1.64 | 0.35 | 99.26 |
| Annotation Cluster 3 | | Enrichment Score: 0.69 | | | |
| Category | Term | Count | % | PValue | FDR |
| GOTERM_BP_FAT | GO:0009991~response to extracellular stimulus | 3 | 0.98 | 0.05 | 50.48 |
| GOTERM_BP_FAT | GO:0008284~positive regulation of cell proliferation | 3 | 0.98 | 0.14 | 88.69 |
| GOTERM_BP_FAT | GO:0042127~regulation of cell proliferation | 3 | 0.98 | 0.37 | 99.84 |
| SP_PIR_KEYWORDS | nucleus | 6 | 1.97 | 0.68 | 100.00 |
| Annotation Cluster 4 | | Enrichment Score: 0.68 | | | |
| Category | Term | Count | % | PValue | FDR |
| SP_PIR_KEYWORDS | phosphotransferase | 3 | 0.98 | 0.03 | 27.99 |
| SP_PIR_KEYWORDS | ATP | 3 | 0.98 | 0.04 | 35.78 |
| UP_SEQ_FEATURE | active site:Proton acceptor | 4 | 1.31 | 0.05 | 47.86 |
| SP_PIR_KEYWORDS | nucleotide-binding | 6 | 1.97 | 0.06 | 52.76 |
| SP_PIR_KEYWORDS | atp-binding | 5 | 1.64 | 0.09 | 66.39 |
| GOTERM_CC_FAT | GO:0045202~synapse | 3 | 0.98 | 0.10 | 68.57 |
| SP_PIR_KEYWORDS | transferase | 5 | 1.64 | 0.10 | 71.77 |
| UP_SEQ_FEATURE | domain:Protein kinase | 3 | 0.98 | 0.12 | 79.48 |
| UP_SEQ_FEATURE | nucleotide phosphate-binding region:ATP | 4 | 1.31 | 0.13 | 80.63 |
| INTERPRO | IPR017441:Protein kinase, ATP binding site | 3 | 0.98 | 0.14 | 79.95 |
| INTERPRO | IPR000719:Protein kinase, core | 3 | 0.98 | 0.15 | 82.41 |
| UP_SEQ_FEATURE | binding site:ATP | 3 | 0.98 | 0.16 | 86.96 |
| GOTERM_MF_FAT | GO:0032555~purine ribonucleotide binding | 6 | 1.97 | 0.17 | 87.03 |
| GOTERM_MF_FAT | GO:0032553~ribonucleotide binding | 6 | 1.97 | 0.17 | 87.03 |
| GOTERM_MF_FAT | GO:0017076~purine nucleotide binding | 6 | 1.97 | 0.19 | 90.55 |
| GOTERM_MF_FAT | GO:0005524~ATP binding | 5 | 1.64 | 0.21 | 93.06 |
| GOTERM_MF_FAT | GO:0032559~adenyl ribonucleotide binding | 5 | 1.64 | 0.22 | 93.72 |
| SP_PIR_KEYWORDS | kinase | 3 | 0.98 | 0.22 | 94.81 |
| GOTERM_MF_FAT | GO:0030554~adenyl nucleotide binding | 5 | 1.64 | 0.25 | 95.89 |
| GOTERM_MF_FAT | GO:0001883~purine nucleoside binding | 5 | 1.64 | 0.26 | 96.40 |
| GOTERM_MF_FAT | GO:0004672~protein kinase activity | 3 | 0.98 | 0.26 | 96.48 |
| GOTERM_MF_FAT | GO:0001882~nucleoside binding | 5 | 1.64 | 0.26 | 96.61 |
| GOTERM_MF_FAT | GO:0000166~nucleotide binding | 6 | 1.97 | 0.29 | 97.96 |
| GOTERM_BP_FAT | GO:0006468~protein amino acid phosphorylation | 3 | 0.98 | 0.30 | 99.25 |
| GOTERM_CC_FAT | GO:0005829~cytosol | 4 | 1.31 | 0.32 | 98.70 |
| GOTERM_BP_FAT | GO:0016310~phosphorylation | 3 | 0.98 | 0.38 | 99.86 |
| GOTERM_CC_FAT | GO:0044430~cytoskeletal part | 3 | 0.98 | 0.42 | 99.80 |
| GOTERM_BP_FAT | GO:0006793~phosphorus metabolic process | 3 | 0.98 | 0.48 | 99.99 |
| GOTERM_BP_FAT | GO:0006796~phosphate metabolic process | 3 | 0.98 | 0.48 | 99.99 |
| GOTERM_CC_FAT | GO:0043228~non-membrane-bounded organelle | 5 | 1.64 | 0.56 | 99.99 |
| GOTERM_CC_FAT | GO:0043232~intracellular non-membrane-bounded organelle | 5 | 1.64 | 0.56 | 99.99 |
| GOTERM_CC_FAT | GO:0005856~cytoskeleton | 3 | 0.98 | 0.62 | 100.00 |
| SP_PIR_KEYWORDS | cytoplasm | 5 | 1.64 | 0.65 | 100.00 |
| GOTERM_CC_FAT | GO:0044459~plasma membrane part | 4 | 1.31 | 0.66 | 100.00 |
| SP_PIR_KEYWORDS | nucleus | 6 | 1.97 | 0.68 | 100.00 |
| Annotation Cluster 5 | | Enrichment Score: 0.56 | | | |
| Category | Term | Count | % | PValue | FDR |
| GOTERM_BP_FAT | GO:0009725~response to hormone stimulus | 3 | 0.98 | 0.12 | 82.89 |
| GOTERM_BP_FAT | GO:0009719~response to endogenous stimulus | 3 | 0.98 | 0.14 | 87.73 |
| GOTERM_BP_FAT | GO:0010033~response to organic substance | 3 | 0.98 | 0.33 | 99.62 |
| GOTERM_CC_FAT | GO:0000267~cell fraction | 3 | 0.98 | 0.49 | 99.95 |
| GOTERM_BP_FAT | GO:0007242~intracellular signaling cascade | 3 | 0.98 | 0.62 | 100.00 |
| Annotation Cluster 6 | | Enrichment Score: 0.37 | | | |
| Category | Term | Count | % | PValue | FDR |
| GOTERM_CC_FAT | GO:0031090~organelle membrane | 4 | 1.31 | 0.22 | 94.08 |
| GOTERM_BP_FAT | GO:0016192~vesicle-mediated transport | 3 | 0.98 | 0.24 | 97.84 |
| GOTERM_CC_FAT | GO:0005829~cytosol | 4 | 1.31 | 0.32 | 98.70 |
| GOTERM_CC_FAT | GO:0044459~plasma membrane part | 4 | 1.31 | 0.66 | 100.00 |
| GOTERM_CC_FAT | GO:0005886~plasma membrane | 6 | 1.97 | 0.70 | 100.00 |
| SP_PIR_KEYWORDS | phosphoprotein | 9 | 2.95 | 0.79 | 100.00 |
| Annotation Cluster 7 | | Enrichment Score: 0.37 | | | |
| Category | Term | Count | % | PValue | FDR |
| SP_PIR_KEYWORDS | glycoprotein | 9 | 2.95 | 0.18 | 90.07 |
| UP_SEQ_FEATURE | glycosylation site:N-linked (GlcNAc...) | 8 | 2.62 | 0.29 | 98.17 |
| SP_PIR_KEYWORDS | membrane | 10 | 3.28 | 0.43 | 99.85 |
| UP_SEQ_FEATURE | transmembrane region | 8 | 2.62 | 0.47 | 99.95 |
| SP_PIR_KEYWORDS | transmembrane | 8 | 2.62 | 0.48 | 99.95 |
| GOTERM_CC_FAT | GO:0016021~integral to membrane | 9 | 2.95 | 0.56 | 99.99 |
| GOTERM_CC_FAT | GO:0031224~intrinsic to membrane | 9 | 2.95 | 0.61 | 100.00 |
| UP_SEQ_FEATURE | topological domain:Cytoplasmic | 5 | 1.64 | 0.67 | 100.00 |
| Annotation Cluster 8 | | Enrichment Score: 0.03 | | | |
| Category | Term | Count | % | PValue | FDR |
| SP_PIR_KEYWORDS | zinc | 3 | 0.98 | 0.79 | 100.00 |
| GOTERM_MF_FAT | GO:0008270~zinc ion binding | 3 | 0.98 | 0.91 | 100.00 |
| SP_PIR_KEYWORDS | metal-binding | 3 | 0.98 | 0.92 | 100.00 |
| GOTERM_MF_FAT | GO:0046914~transition metal ion binding | 3 | 0.98 | 0.96 | 100.00 |
| GOTERM_MF_FAT | GO:0046872~metal ion binding | 4 | 1.31 | 0.98 | 100.00 |
| GOTERM_MF_FAT | GO:0043169~cation binding | 4 | 1.31 | 0.98 | 100.00 |
| GOTERM_MF_FAT | GO:0043167~ion binding | 4 | 1.31 | 0.99 | 100.00 |
